# Pharmacogenetics of BCR/ABL Inhibitors in Chronic Myeloid Leukemia

**DOI:** 10.3390/ijms160922811

**Published:** 2015-09-21

**Authors:** Marialuisa Polillo, Sara Galimberti, Claudia Baratè, Mario Petrini, Romano Danesi, Antonello Di Paolo

**Affiliations:** 1 Department of Clinical and Experimental Medicine, Section of Pharmacology, University of Pisa, Via Roma 55, 56126 Pisa, Italy; E-Mails: polmarilu@yahoo.it (M.P.); romano.danesi@unipi.it (R.D.); 2 Department of Clinical and Experimental Medicine, Section of Hematology, University of Pisa, Via Roma 57, 56126 Pisa, Italy; E-Mails: sara.galimberti@med.unipi.it (S.G.); claudia.barate@gmail.com (C.B.); mario.petrini@med.unipi.it (M.P.)

**Keywords:** pharmacogenetics, pharmacogenomics, chronic myeloid leukemia, imatinib, nilotinib, dasatinib, bosutinib, ponatinib, transmembrane transporter

## Abstract

Chronic myeloid leukemia was the first haematological neoplasia that benefited from a targeted therapy with imatinib nearly 15 years ago. Since then, several studies have investigated the role of genes, their variants (*i.e*., polymorphisms) and their encoded proteins in the pharmacokinetics and pharmacodynamics of BCR-ABL1 tyrosine kinase activity inhibitors (TKIs). Transmembrane transporters seem to influence in a significant manner the disposition of TKIs, especially that of imatinib at both cellular and systemic levels. In particular, members of the ATP-binding cassette (ABC) family (namely ABCB1 and ABCG2) together with solute carrier (SLC) transporters (*i.e*., SLC22A1) are responsible for the differences in drug pharmacokinetics. In the case of the newer TKIs, such as nilotinib and dasatinib, the substrate affinity of these drugs for transporters is variable but lower than that measured for imatinib. In this scenario, the investigation of genetic variants as possible predictive markers has led to some discordant results. With the partial exception of imatinib, these discrepancies seem to limit the application of discovered biomarkers in the clinical settings. In order to overcome these issues, larger prospective confirmative trials are needed.

## 1. Introduction

Patients affected by chronic myeloid leukemia (CML) were the first ones who benefited from a targeted therapy with imatinib in the late 1990s [1] and this drug remains the most frequently used as first-line therapy for CML [2]. Nowadays several targeted agents are in clinical use to treat haematological and solid neoplasms, while a multitude of them are already in clinical development. Therefore, after the introduction of tyrosine kinase activity inhibitors (TKIs), the outcome of CML patients has improved, with 82% having complete cytogenetic responses (CCyR), and more than 90% of patients alive and free from progression eight years after the diagnosis [3,4,5,6].

Nevertheless, about 30% of the treated patients must interrupt the treatment because of poor tolerability or cytogenetic or molecular failure [7,8]. Those situations may be managed by the substitution of imatinib with second or third-generation TKIs, such as nilotinib, dasatinib, bosutinib and the most recent ponatinib. Although they share with imatinib the same mechanisms of action, the spectrum of their inhibitory capabilities against other intracellular kinases is different, as well as their substrate affinity with respect to metabolic enzymes and transmembrane transporters. As a consequence, there are non-negligible differences among the TKIs and this means that the predictive biomarkers for one drug do not perfectly fit for another one.

Therefore, the present review will present and discuss possible pharmacogenetic markers predictive of response to TKIs. The published literature has been evaluated through the PubMed database using the following keywords in different combinations: chronic myeloid leukemia, imatinib, nilotinib, dasatinib, bosutinib, ponatinib, pharmacogenetic, pharmacogenomic, polymorphisms, gene expression, pharmacokinetics. Other pertinent articles have been selected among the references of retrieved literature.

## 2. Many Pieces for the Pharmacogenetic Puzzle

Many preclinical experiments and clinical trials have focused their efforts to investigate any possible gene variation (in its broadest meaning) that could be predictive of treatment efficacy or toxicity and that could be applied in the clinical practice. Different studies identified at the same time more than one pharmacogenomic determinant, hence revealing the multifactorial characteristics of TKI efficacy and/or toxicity. In other cases, the search for predictive biomarkers was conducted by the aid of a pharmacokinetic approach, through the analysis of plasma concentrations of drugs. In this search for biomarkers, a clear-cut definition of clinical endpoints is an essential requirement. In CML patients, the treatment efficacy may be graded across a hematologic, cytogenetic and molecular response, according to the most recent European guidelines [7]. In particular, molecular response may be scored depending on the magnitude of the logarithmic decrease of the BCR-ABL1 transcript (MR^3^ = major molecular response [MR]; MR^4^/MR^4.5^/MR^5^ = deep or complete molecular response) and according to the time required to attain these responses [7].

The above-listed aspects of the TKI investigation may represent some critical issues in clinical trials, while they may explain both discrepancies among different studies and the lack of complete, matched associations between the presence of certain polymorphisms and clinical efficacy.

### 2.1. Liver Enzymes

Initial studies confirmed the involvement of CYP3A isoforms in the metabolic clearance of imatinib [9], whose main metabolite is *N*-desmethyl-imatinib (CPG74588). The latter accounts for approximately 20% of plasma levels of the parent drug and its cytotoxic effect as an active metabolite is estimated to be approximately 3–4 times lower than that of imatinib [10]. Because imatinib is a substrate of liver enzymes, it is subjected to drug-drug interactions. In particular, the intake of rifampin, a hepatic CYP3A inducer, causes a significant decrease in the systemic exposure of the drug (approximately 68%) [11], whereas the administration of ketoconazole, an inhibitor of liver enzymes, led to a 40% increase in exposure [12].

Further evidence strengthened the role of CYP isoforms as determinants of the imatinib response. The use of a probe drug such as quinidine allowed the stratification of patients according to their systemic CYP3A activity [13], and the complete MR was significantly associated with the highest *in vivo* enzymatic activity. This result suggested that biotransformation of imatinib by CYP3A led to the active metabolite. Several clinical trials collected evidence about the association between CYP3A4 and CYP3A5 polymorphisms and response to imatinib [14,15]. It is worth noting that in all these studies the evaluation of CYP genotypes was part of a wider investigation, which often included transmembrane transporters.

The CYP3A4 isoform catalyses the biotransformation of the more recent TKIs dasatinib and ponatinib into active metabolites that have an equipotent or a 4-time lower inhibitory activity in comparison to the parent drug, respectively [16,17]. Conversely, nilotinib and bosutinib are inactivated by that isoform. Interestingly, no polymorphisms of the CYP3A4/5 genes have been investigated with respect to changes in the pharmacokinetics or pharmacodynamics of these TKIs, whereas the major efforts have been addressed to the evaluation of potential drug-drug interactions [18,19,20].

### 2.2. Transmembrane Transporters

In order to bypass the possible negative effect exerted by the transmembrane transporters on TKI efficacy, researchers have recently designed additional drugs whose efficacy is not significantly affected by the ATP-binding cassette (ABC) transporters, as is the case for ponatinib [21]. The importance of the ABC and solute carrier (SLC) transporters relies on their variable expression on the membrane of different cell types, their wide distribution within the organism and their involvement in the cellular influx or efflux of the drugs.

#### 2.2.1. ABCB1

Among the most investigated transporters, a prominent role is played by the ABCB1 (Table 1). Since the first pharmacokinetic/pharmacogenetic studies, it was evident that this protein is involved in the extrusion of imatinib outside the Philadephia + leukemic cells [22,23]. In particular, the ABCB1 overexpression has been associated with resistance to imatinib [22,24], its reduced intracellular concentration [25], and a diminished inhibition of BCR-ABL1 [26]. Furthermore, the distribution of this transporter on the membrane of the epithelial cells in the gut mucosa and excretory organs [27] is responsible for a lower tissue exposure to imatinib and is considered as a predictive marker of drug response. In particular, those patients carrying minor alleles for the c.1236C>T and c.2677G>T/A single nucleotide polymorphisms (SNPs) experienced a better benefit from imatinib, whereas the 1236C-2677G-3435C haplotype was associated with less frequent MR [28,29]. On the other hand, patients homozygous for the low-activity c.1236T allele had the highest plasma concentrations of imatinib. Therefore, all these observations show that the transporters’ activity could act at two different levels: a highest ABCB1 activity causes a reduced intestinal absorption (*i.e.*, diminished bioavailability) and increases excretion of imatinib from kidney and liver, while at the cellular level it causes a lower cytoplasmic accumulation of the drug, thus reducing the BCR-ABL1 inhibition capability.

**Table 1:** Clinical trials investigating the role of ABCB1 and ABCG2 on the imatinib efficacy. In the case of ABCB1, all of the listed studies evaluated the c.1236C>T, c.2677G>T/A, c.3435C>T polymorphisms. Other SNPs (single nucleotide polymorphisms) are indicated.

Transporter	SNPs *	Patients	Main Results	Reference
ABCB1	c.-129T>C	189	c.3435CT/TT was an adverse genotype for complete MR in Caucasians ^a^	[15]
		215	c.1236CC and CGC haplotype were associated with resistance, while c.2677TT/TA/AA were related with better CCyR	[30]
		100	TGT haplotype was associated with worse therapeutic effect from imatinib	[31]
		90	c.1236TT and c.2677TT/TA were associated with better major MR rate	[28]
		90	CGC haplotype associated with less frequent major MR	[28]
		52	c.1236TT or c.3435CT/TT were associated with higher resistance; patients with the c.2677AG/AT/AA genotype had better CCyR than those carrying c.2677TT/GT/GG	[32]
		84	c.3435TT associated with significantly longer times to major MR compared to CC/CT genotypes	[33]
		28	Polymorphic alleles were associated with a reduced *ex vivo* ABCB1 activity; the highest transporter activity was present in patients who did not achieve major MR	[29]
ABCG2	c.34G>A	229	c.34GG genotype was associated with lowest rates of major MR and CCyR	[14]
	c.34G>A, c.421C>A	215	c.421CC associated with resistance; AA haplotype, better response	[30]
	c.421C>A	82	c.421CC/CA associated with lower rate of major MR ^b^	[34]

*, other than c.1236C>T, c.2677G>T/A, c.3435C>T; ^a^, other investigated genes: CYP3A4, CYP3A5, OATP1A2; ^b^, further genes investigated: CYP3A4, CYP3A5, CYP2C9, CYP2C19, CYP2D6, ABCB1, SLC22A1 and SLC22A2. Abbreviations: MR, molecular response; CCyR, complete cytogenetic response.

However, several preclinical and clinical studies reported discordant results about the relationship between the ABCB1 activity and efficacy of imatinib. In the K562 cell line, the expression of ABCB1 variants was not associated with increased resistance against imatinib [35] while the c.1236T-c.2677T-c.3435T haplotype was associated with the highest ABCB1 expression on cell membranes. Among clinical trials, Ni and coworkers [32] found that the resistance to imatinib was more frequent in c.1236TT and c.3435TT or CT patients; the same conclusion was sustained by Ali and colleagues [31]. Furthermore, Vine and colleagues showed that the time to major MR was significantly longer in patients harbouring the c.3435TT genotype than in subjects carrying the CC or CT genotypes [33]. Moreover, although the c.1236C-c.2677G-c.3435C haplotype was significantly related to an increased risk of resistance, the c.2677T/A variant was associated with a lower MR rate in another recent study [30].

In order to better clarify the effect of the ABCB1 SNPs in imatinib pharmacokinetics, patients’ genotypes and haplotypes were investigated also by mathematical models including population pharmacokinetic approaches [36]. Results from two independent studies on 67 and 60 Caucasian subjects excluded a significant influence of the ABCB1 polymorphisms on the drug pharmacokinetics [37,38]. On the contrary, a third trial found a significant association among a combined ABCB1/SLC22A1 haplotype, imatinib clearance, and plasma concentrations [39]. However, the latter study enrolled only 38 Asian patients and imatinib clearance was calculated on the basis of trough plasma concentrations [39]. Therefore the discrepancies among these studies could depend on the different number of enrolled patients, their race (Caucasian *vs.* Asian subjects), and the employed methodologies. In another study, patients who were homozygotes at the three loci for the polymorphic alleles (*i.e*., c.1236TT, c.2677TT and c.3435TT) had a higher imatinib clearance; interestingly, these patients reduced imatinib dose for toxicity less frequently than the other ones [40]. That “apparent paradox” was explained by different mechanisms (*i.e*., a more significant inhibition of ABCB1 activity by imatinib in c.3435CC patients, or the presence of other tagged SNPs), but the Authors concluded that confirmatory trials were needed.

In the case of the second-generation TKIs, a different affinity toward ABCB1 has been reported. *In vitro* experiments demonstrated that nilotinib is not an ABCB1 substrate [25,41], and this TKI could also inhibit the activity of the transporter [42,43,44]. Moreover, imatinib, nilotinib and bosutinib are “comparatively weaker ABCB1 substrates” with respect to *N*-desmethyl-imatinib and dasatinib [35]. However, results are still contradictory: other two *in vitro* studies demonstrated that nilotinib is a substrate of ABCB1 [26] and that the increased expression of the transporter is associated with a reduced sensitivity of the K562 leukemia cell line to this TKI [45]. Finally, a recent population pharmacokinetic study demonstrated that the excretion of nilotinib was influenced by the c.2677G>T/A SNP [46], but further clinical trials are required to confirm these data.

Also dasatinib is a substrate of ABCB1 [35,41,47], as showed by the recovery of its efficacy when resistant cells were exposed to PSC833, a specific ABCB1 inhibitor [48]. Analogously, a murine model, knock-out for ABCB1 and/or ABCG2, confirmed that dasatinib is an ABCB1 substrate [49]. However, only one *in vitro* study demonstrated that the use of ABCB1 inhibitors seems not to influence intracellular concentrations of dasatinib in CML-CD34(+) progenitors [47]. Those preclinical results strongly suggest that dasatinib is effectively a substrate of ABCB1, but the relative scarcity of clinical trials does impede the further evaluation of those data.

Concerning bosutinib, some authors reported that it is a weak substrate of ABCB1 [35]. Further *in vitro* experiments on the K562 cell line overexpressing the drug transporters ABCB1, ABCG2, and SLC22A1 showed that only ABCB1 was responsible for the active bosutinib transport, because resistance was overcome by the addition of the ABCB1 inhibitor verapamil [50].

Finally, some authors reported that ponatinib is able to reverse the ABCG2- and ABCB1-mediated multi-drug resistance [51]. However, a more recent study seems not to confirm those results for ponatinib [21].

Overall, the sometime contradictory results can be explained by (a) the relatively lower number of patients treated with second-generation TKIs in respect to those receiving imatinib; (b) the lack of standardized methods and instruments for the measurement of plasma concentrations of TKIs; and (c) the adoption of variable modelling approaches.

#### 2.2.2. ABCG2

The second transmembrane transporter that caught the attention of the researchers is ABCG2, formerly known as breast cancer resistance protein (BCRP). Several *in vitro* studies demonstrated that imatinib is a substrate of this transporter [23,24,25,52,53], and that imatinib is able to inhibit the transporter activity [54]. Therefore, preclinical studies suggested that the variable ABCG2 activity could influence the steady-state plasma concentrations of the drug and, ultimately, its efficacy [55]. That hypothesis has been confirmed by several clinical trials: the high-activity of the c.421CC and c.34GG ABCG2 genotypes has been associated with lower plasma concentrations [56], whereas the c.421C>A SNP was predictive of significant changes in the apparent clearance of imatinib [37]. Furthermore, c.421C>A and c.34G>A genotypes predicted a lower rate of complete cytogenetic response to imatinib [14,30]. Interestingly, a study enrolling 154 CML patients found that the c.421C>A SNP was an independent predictor of complete MR in multivariate analysis (OR = 0.3953, *p* = 0.0284, CC as reference) [57]. Another trial demonstrated that patients carrying the c.421AA genotype achieved the major MR in a greater percentage (*n* = 4, 100%) compared to individuals with other genotypes (*n* = 73, 12.3%) [34], but the unbalanced number of patients between the two groups of this study should be taken into consideration. Finally, the cumulative incidence of major MR was the same in patients who received imatinib 600 or 400 mg/day, if the latter ones carried a favourable G-G haplotype composed by two SNPs (rs12505410 and rs2725252) of the ABCG2 gene [58]. Overall, all these clinical trials show that ABCG2 SNPs are predictive markers of imatinib efficacy and pharmacokinetics.

Controversial results have been published regarding the ABCG2 affinity and second-generation TKIs. Nilotinib [24,41,53], dasatinib [41,48] and ponatinib [51] are substrate of this transporter, even if nilotinib and ponatinib are also capable of inhibiting the transporter activity [42,51,59]. However, an *in vivo* study with knock-out mice suggested a partial role for ABCG2 in the transmembrane transport of dasatinib [49], while other *in vitro* experiments failed to confirm a role for ABCG2 in drug disposition [25,47]. Finally, a recent work denies a role for ABCG2 as a transporter of ponatinib [21]. At present, preclinical investigations seem unable to offer robust and reliable data, and clinical studies investigating the role of ABCG2 on the second-generation TKIs efficacy are necessary to draw conclusive considerations.

#### 2.2.3. SLC22A1

SLC22A1, also known as hOCT1 (human organic cation transporter member 1), plays an important role in cellular uptake of imatinib [25,60,61]. As a consequence, the different cellular uptake and cytoplasmic retention of the drug are significantly associated to the variable BCR-ABL1 inhibiting activity, which in turn is correlated with cytogenetic and molecular responses [62] (Table 2). Furthermore, SLC22A1 gene harbours several polymorphisms that are associated with an altered transport activity [63]. Thus, several studies evaluated the correlations between its polymorphisms and response to imatinib: the SLC22A1 SNP c.1022C>T and a combination of polymorphisms were predictive of higher rates of major cytogenetic and molecular responses [15]. Analogously, the c.480CC genotype was significantly associated with a higher percentage of event-free survival (EFS) at 24 months with respect to the CG or GG genotypes [38].

**Table 2:** Studies evaluating the possible correlation between clinical endpoints and polymorphisms of SLC22A1, OCTN1, OATP1A2 and CYP3A5 genes, their mRNA levels or transporter activity in CML patients treated with imatinib.

Genes	Polymorphisms, Gene Expression, Functional Test	Patients	Main Results	Reference
*SLC22A1*	c.1002C>T	189	c.1022CT/TT were adverse genotypes for major cytogenetic response in all patients	[15]
	c.1260-1262delGAT, c.1222>G ^a^	336	Deletion was associated with time to treatment failure, but it was restored by the c.1222G allele	[64]
	c.480G>C	229	c.480GG associated with high rate of loss of response or treatment failure	[14]
	c.1260-1262delGAT, c.1222A>G	153	c.1222AA/AG genotypes associated with longer time to MR	[65]
	Gene expression	28	Higher levels of mRNA were observed in patients who achieved major and complete MR	[29]
	Gene expression	70	Highest pre-treatment mRNA levels were associated with better CCyR rates, PFS and OS ^b^	[61]
	Transporter activity	-	High TA, better major MR at 60 months; low TA, lower OS, EFS and higher kinase domain mutation rate	[66]
	Haplotype ^c^	189	Associated with both complete and major MR	[15]
*OCTN1*	c.1507C>T	189	c.1507TT was an adverse genotype for major MR in all patients and in Caucasians	[15]
*SLC22A1, OCTN1, OATP1A2*	various	189	Complete and major MR were associated with a combination of SNPs	[15]
*CYP3A5*	g.12083G>A	229	g.12083AA genotype was associated with lowest rates of major MR and CCyR	[14]

^a^, further 21 polymorphisms have been investigated, but they were not related to the clinical outcome; ^b^, pretreatment mRNA levels of ABCB1, ABCG2 and ABCC1 did not predict treatment outcome; ^c^, combined molecular endpoint composed by 4 SLC22A1 SNPs. Abbreviations: MR, molecular response; CCyR, complete cytogenetic response; OS, overall survival; EFS, event-free survival; TA, transporter activity.

The c.1260-1262delGAT polymorphism (M420del) was associated with an increased risk of treatment failure with imatinib [64], but that risk would be counterbalanced by the concomitant presence in *cis* of the c.1222A>G SNP. This still remains a debated issue, because other authors never found the M420del polymorphism in *cis* with the c.1222A>G SNP [65]. Similar results have been obtained by Tzetkov and coworkers who found that the M420del polymorphism was responsible for the loss in the substrate specificity “*that is only marginally affected by M408V*” (*i.e*., c.1222A>G) [67]. Beyond the discrepancies observed in these trials, two important points of discussion emerged: first, the c.1222A>G polymorphism is responsible for an alternative splicing, and it is associated with a longer time to achieve an optimal response [65]. Second, the differences in SLC22A1 mRNA levels could depend on the primers used for the real-time PCR [64], thus explaining the contrasting results obtained when the relationship between SLC22A1 gene expression levels and the clinical outcome was assessed [61,65,68,69,70].

As reported about ABCB1, population pharmacokinetic analyses were planned to evaluate the role of SLC22A1 in imatinib disposition. The rationale for this approach resides in tissue expression of the transporter, because SLC22A1 is present in the gut mucosa and the liver where it would participate in the absorption and excretion of imatinib, respectively [27,71,72]. Some authors reported that this transporter does not influence the drug pharmacokinetics in CML patients carrying low-activity variants of the transporter [73]. On the contrary, two recent independent trials confirmed the relationship among the SLC22A1 polymorphisms, plasma concentrations, and imatinib systemic clearance [38,39]. In particular, the presence of the c.480CA/AA genotypes was associated with a drop of 12% in drug clearance, whereas the mean trough plasma concentrations increased by 45% compared to the c.480CC individuals [38]. Furthermore, this genetic variation explained approximately 10% of the inter-individual variability of imatinib pharmacokinetics [38].

Recently, two research teams published interesting findings about the possibility of adopting SLC22A1 as a potential marker of the imatinib effect. In the first study, a low transport activity in peripheral mononuclear cells was associated with poor response rates and the highest probability of transformation to accelerated phase or blast crisis [66]. Therefore, the study proposed an increase of daily dose of imatinib to overcome the low activity of SLC22A1 [74]. Intriguingly, the presence of some polymorphisms, namely c.181C>T, c.1260_1262delGAT, and c.1222A>G, were not related to the SLC22A1 activity nor to the achievement of the major MR at 24 months [75]. Another *in vitro* study from Nies and colleagues clearly demonstrated that the uptake and the cytotoxic effect of imatinib in CML cell lines and patients’ leukemic cells were independent from the SLC22A1 expression [76]. Interestingly, an SLC22A1-knockout murine model did not show any alteration of the plasma kinetics and liver concentrations of imatinib compared to the controls [76]. Overall, the comparison of those findings with previously obtained results [62,74] suggested that some transporters other than SLC22A1 could be involved in imatinib uptake, as already hypothesised by Hu and colleagues [73].

The SLC22A1 transporter does not recognize nilotinib as a substrate [25,62,77] nor dasatinib [78], as it has been also demonstrated in peripheral mononuclear cells obtained from chronic-phase CML patients [48]. However, the mathematical modelling through a factor analysis of mixed data demonstrated that the hOCT1 c.480C>G genotype could be a possible covariate of the pharmacokinetic model of nilotinib, and c.480CC patients had a significantly better three-year EFS with respect those individuals carrying at least one c.480G polymorphic allele [46]. As pointed out by the authors, these results shed light on the role of the SLC22A1 transporter for nilotinib, but further studies are still required for conclusive considerations.

In summary, preclinical and clinical results are strengthening the crucial role of SLC22A1 on imatinib kinetics at both the systemic and cellular level, despite some studies reported contrasting results that should be taken into consideration. Beyond this issue, the question regarding which SLC22A1 polymorphism is the best predictive biomarker of imatinib efficacy or whether a functional assay could be better than pharmacogenetic analyses is still unanswered. In the case of second-generation TKIs, data published in the literature are scarce for both preclinical experiments and clinical trials, and a definitive relationship between these drugs and SLC22A1 may not be demonstrated nor excluded. It is likely that an increasing number of studies evaluating nilotinib, dasatinib, and other most recent TKIs could solve this issue.

#### 2.2.4. Other Transmembrane Transporters

The expression of other transmembrane transporters on different tissues may account for the unexplained inter-individual variability in both pharmacokinetics and pharmacodynamics. The uptake of several TKIs, other than those directed against BCR-ABL1, was clearly increased by the expression of the SLCO1B members in the human embryonic kidney cell line HEK293 [79]. In particular, imatinib, nilotinib, and dasatinib accumulated within the cells when SLCO1B3 was expressed, but for nilotinib a significant increase was observed also in the presence of SLCO1B1. Recent results from a clinical trial have demonstrated that the SLCO1B3 polymorphism c.334T>G significantly influenced the absorption of nilotinib in CML patients [46], because the bioavailability of the drug was increased by 11% in patients with c.334TT genotype. Moreover, those individuals carrying the c.521C allele of SLCO1B3 had a longer time to achieve both the CCyR and the major MR. These results suggest that the activity of the transporter is highly associated with the absorption process, but confirmative multicentre trials are still needed.

The involvement of other transmembrane transporters in TKI disposition is still under investigation. In particular, imatinib was demonstrated to be a substrate of ABCC4 and SLCO1A2 in a clinical trial [73], whereas another study confirmed the influence of SLC22A4 but not of SLCO1A2 on the MR [15].

Therefore, the relationship between imatinib and the three transmembrane transporters SLC22A1, ABCB1, and ABCG2 seems the most promising one to be translated into the clinical practice, but prospective confirmative trials are still needed. At present, the data for second-generation TKIs are discordant and insufficient to identify the best candidate among the different transporters (with their polymorphisms), with the exception of ABCB1 for dasatinib. An improved knowledge about the relationship between BCR-ABL1 inhibitors and transporters is an urgent need, because nilotinib and dasatinib are registered agents for first-line therapy [80], and they represent an effective second- or third-line treatment for CML patients who fail imatinib [81].

## 3. Discussion

Previous paragraphs describe the role of pharmacogenetics to predict the treatment efficacy (and perhaps tolerability) in CML patients: among the possible proteins involved in the kinetics of TKIs at the cellular and systemic level, transmembrane transporters play a pivotal role because they influence both intracellular and plasma concentrations of the drugs. In turn, the variable concentrations of imatinib, nilotinib, and other TKIs may result in significant variations in efficacy and tolerability.

Among the different transporters, ABCB1 participates in the drug extrusion and excretion at the cellular level, but it could influence also the intestinal absorption because its characteristic tissue distribution [27]. On the other side, ABCB1 is a well-known protein, it recognizes a number of pharmacological substrates and its gene harbours several polymorphisms [82,83], making the transporter an optimal biomarker for the prediction of imatinib efficacy [84]. Interestingly, the activity of the transmembrane transporters could be modulated by specific inhibitors to overcome treatment failure [85], but in one report the ABCB1 inhibition did not enhance the efficacy of imatinib [86]. About nilotinib, preclinical data are sometimes discordant and there is only one clinical study showing the involvement of ABCB1 in nilotinib disposition [46]. Among the other TKIs, dasatinib displays a sufficient substrate affinity for ABCB1 for which the transporter (and its polymorphisms) may represent a pharmacogenetic biomarker [35].

The efflux of imatinib from the cells is dependent also on the presence/activity of ABCG2 [23,24], whose polymorphisms have been associated with the clinical efficacy of the drug [25,57]. Whether the transporter is also involved in the kinetics of the second-generation TKIs is not still clearly demonstrated nor excluded because of the controversial results obtained until now [25,51].

The introduction of TKIs in clinical settings addressed researchers’ attention toward those transporters that ensure drug uptake within leukemic cells. Several studies confirm the role of SLC22A1 in the cellular uptake of imatinib and, as a consequence, the inhibition of BCR-ABL1 activity. In fact, clinical endpoints, such as cytogenetic or molecular responses, are predicted by polymorphisms of SLC22A1, but also tolerability and changes of drug disposition may be anticipated by the evaluation of allelic variants of the gene. These observations are based on tissue distribution of the transporter, being capable of influencing the drug excretion and/or absorption [27]. However, one study demonstrated that imatinib uptake does not depend on SLC22A1 in different *in vitro* and *in vivo* models [76], suggesting that other transporters could be involved despite “the mechanisms responsible for imatinib uptake into leukemic cells are still elusive” [76].

TKIs other than imatinib, namely dasatinib and nilotinib, can inhibit members of the SLC22A family [87], and preliminary data pointed out at SLC22A1 as a nilotinib transporter [46], but the evidence is still limited.

Overall, the landscape of TKI pharmacogenetics is characterized by several points of interest, such as the relationships among gene polymorphisms, altered pharmacokinetics, and drug effects. A number of factors suggest that pharmacogenetic results could be reliable for the prediction of treatment efficacy, but the discrepant data limit the confidence and the application of the biomarker (Table 3). The reasons for these unexpected results may depend on the biologic system used (*i.e.*, different cell lines) [73], the range of drug concentrations evaluated [25,41], or the interplay among different genes and polymorphisms, the majority of which are not investigated at the same time. Population pharmacokinetic analyses and pharmacokinetic/pharmacodynamics mixed models could overcome some of these issues, at least in part, because each covariate (*i.e*., each polymorphism) is introduced within the model that in turn quantifies the kinetic and/or dynamic influence of the factor on the whole system (*i.e*., cells or individuals).

**Table 3:** Issues, causes and possible solutions related to contrasting results published in the literature regarding the search for TKI biomarkers.

Study Phase	Issues	Causes	Possible Solutions
Preclinical studies	Discrepancies in substrate affinity/role of transporters in TKIs kinetics at both the cellular and systemic level	Differences in cell lines, in *in vivo* models, and range of drug concentrations used	Adoption of homogenous *in vitro* and *in vivo* models Same experimental conditions
Clinical Trials	Contradictory results reported for the evaluation of the relationship between pharmacogenetic results and treatment outcome	Gene candidate strategy with limited number of genes/polymorphisms Definition of clinical endpoints has been changed over time Patients’ compliance and adherence have not been fully investigated	Increase the number of genes/polymorphisms investigated at one time Strict adherence to clinical guidelines for endpoints definition Check for patients’ compliance

Another possible confounding factor could be the patient’s adherence to the prescribed treatment. The problem of patients’ compliance has been clearly examined in the recent past [69], and the findings demonstrated that clinical efficacy depends in large part on the regular intake of the drugs. Therefore, the assessment of adherence becomes a prerequisite for interpreting clinical results, and the therapeutic drug monitoring of plasma TKI concentrations could serve both as a measure for this endpoint [88] and to obtain data for pharmacokinetic analyses [38].

Two questions remain to be answered: when pharmacogenetic biomarkers should be used and what is needed to transfer those markers in clinical settings. Biomarkers may have a rational position in the management of CML patients before the start of the treatment. Pharmacogenomic tests may help in the choice of the TKI according to the patient’s genotype of transmembrane transporters and substrate affinity of drugs, but also the dose could be a matter of choice. When treatment has begun, deepest genetic analyses could be useful to investigate possible unexplained relationships between drugs and toxicities or therapy failures. This approach will certainly benefit from next-generation sequencing and whole genome association studies.

The second answer is a critical issue for many biomarkers in oncology and beyond. Confirmatory prospective clinical trials, which should be aimed at the final validation of the biomarkers, are lacking and the uncertainty due to discrepancies among studies remains an insurmountable hurdle to set up companion diagnostics. A part of the problem is related to the choice of clinical and molecular endpoints (*i.e*., complete cytogenetic response, major or complete MR), but their application according to international guidelines will represent an excellent solution, especially for those biomarkers that should predict the earliest drug effects.

## 4. Conclusions

In conclusion, the investigation of TKI pharmacogenetics in CML patients has led to important results, with the aim to decipher the relationships between genes and drugs. Therefore, a great part of the inter-individual variability could be due to the transmembrane transporters, whose activity may be evaluated by both genetic analyses, and functional tests [75]. However, a part of that variability still remains unexplained, even if other approaches and areas of research (*i.e*., epigenetics) could lead to interesting results in the close future [89]. It is likely that a clear-cut definition of endpoints will bring to more generalizable results, which in turn will help in the setting up of prospective trials.
